# Learning of a mimic odor combined with nectar nonsugar compounds enhances honeybee pollination of a commercial crop

**DOI:** 10.1038/s41598-021-03305-9

**Published:** 2021-12-14

**Authors:** M. Cecilia Estravis-Barcala, Florencia Palottini, Walter M. Farina

**Affiliations:** 1 Laboratorio de Insectos Sociales, Departamento de Biodiversidad y Biología Experimental, Facultad de Ciencias Exactas y Naturales, Buenos Aires, Argentina; 2 Instituto de Fisiología, Biología Molecular y Neurociencias (IFIBYNE), CONICET-Universidad de Buenos Aires, Buenos Aires, Argentina

**Keywords:** Ecology, Neuroscience, Systems biology, Zoology, Ecology, Environmental sciences

## Abstract

The increasing demand on pollination services leads food industry to consider new strategies for management of pollinators to improve their efficiency in agroecosystems. Recently, it was demonstrated that feeding beehives food scented with an odorant mixture mimicking the floral scent of a crop (sunflower mimic, SM) enhanced foraging activity and improved recruitment to the target inflorescences, which led to higher density of bees on the crop and significantly increased yields. Besides, the oral administration of nonsugar compounds (NSC) naturally found in nectars (caffeine and arginine) improved short and long-term olfactory memory retention in conditioned bees under laboratory conditions. To test the effect of offering of SM-scented food supplemented with NSC on honeybees pollinating sunflower for hybrid seed production, in a commercial plantation we fed colonies SM-scented food (control), and SM-scented food supplemented with either caffeine, arginine, or a mixture of both, in field realistic concentrations. Their foraging activity was assessed at the hive and on the crop up to 90 h after treatment, and sunflower yield was estimated prior to harvest. Our field results show that SM + Mix-treated colonies exhibited the highest incoming rates and densities on the crop. Additionally, overall seed mass was significantly higher by 20% on inflorescences close to these colonies than control colonies. Such results suggest that combined NSC potentiate olfactory learning of a mimic floral odor inside the hive, promoting faster colony-level foraging responses and increasing crop production.

## Introduction

The production of approximately 70% of the leading single crops, accounting for up to 35% of global food production, increases with animal pollination and mainly relies on the managed honeybee *Apis mellifera*^1^. Despite the continuously increasing agricultural dependence on pollinators, the global stock of honeybee colonies does not meet current crop pollination demands^2,3^. Such circumstances have led to consider new strategies for the management of pollinators to improve their efficiency in agroecosystems.

Olfactory memories play an important role in honeybees foraging behavior, guiding them towards the learned stimuli^4–7^. Central place foragers, like honeybees, can learn floral odors not only when visiting rewarding flowers but also inside the nest, when successful foragers return to the hive. Such individuals can perform a behavior known as the waggle dance which attracts nestmates that apart from decoding the spatial information, perceive the floral odors impregnated onto the foragers’ body, learning about profitable sources^5^. Also, the distribution of the collected nectar among nestmates through mouth-to-mouth contacts (trophallaxis) can lead to associative learning of floral odors by bees of different ages and consequently, bias colony-foraging decisions^8–10^. Moreover, the offering of scented food inside the hive can also lead to olfactory learning^7,8,11,12^.

Recently, a sunflower mimic (SM) odor that bees generalized with the natural floral scent of this highly pollinator-dependent crop has been developed^13^. Across different experimental approaches, this study shows that feeding colonies a rewarding mimic odor allowed bees to establish olfactory memories that conditioned them to forage on the sunflower crop. This was evinced by higher foraging activity, increased recruitment towards the target inflorescences and reduced delays in waggle dance onset. Moreover, higher densities of bees foraging on the crop were found near colonies fed scented food, and significant gains in yield were observed in different sunflower hybrids.

In addition to odors, attraction and fidelity to a particular flower type can be enhanced by nonsugar constituents (NSC) present in floral rewards, including amino acids, lipids, antioxidants and non-volatile secondary metabolites^14–16^. Such NSC play an important role in plant-pollinator interactions and have been shown to influence pollinator behavior and fitness^16–21^. The alkaloid caffeine and the essential amino acid arginine are present in nectars of many plants exploited by pollinators^15,22^. Caffeine can have a positive effect on honeybee olfactory associative learning, promoting stable and long-term memories (LTM)^23^. This substance can also induce honeybees foraging and recruiting behaviors^24,25^. On the other hand, arginine is commonly found in the nectars and pollens of a wide variety of plants^21,26–29^. In honeybees, it is involved in the synthesis of nitric oxide (NO) an important signaling molecule in LTM formation and other cellular processes^30,31^. Furthermore, the administration of arginine in a concentration of 0.001 mM can affect short-term memory (STM) processing in bees^16,32^.

Despite the co-occurrence of a wide variety of NSC in floral nectars, few studies have investigated the effect of mixtures in pollinator behavior^19^. The oral administration of caffeine and arginine, presented in combination and in concentrations within or below the natural range found in nectars, has recently been reported to have a positive effect in the memory formation processes, improving short and long-term olfactory memory retention in conditioned bees under laboratory conditions^33^. Additionally, this mixture triggered a significant survival rate in the conditioned bees. To our knowledge, there are no prior studies concerning the role of combined NSC and odors in pollinator behavior in the field.

In the present field study, we report the effect of offering of SM-scented food supplemented with NSC on the pollination service provided by honeybees in a sunflower plantation for hybrid seed production. In this agroecosystem, bees are essential to ensure pollination, transferring pollen from male fertile (MF) to male sterile (MS) sunflowers. We fed colonies SM-scented food (as a control), and SM-scented food supplemented with either caffeine, arginine, or a mixture of both, in field realistic doses. We assessed honeybee foraging activity in terms of colony activity and density of foragers on the crop. Additionally, to evaluate whether the effect of the treatments translated into an increase in yield, we estimated the seed set and seed mass of sunflower inflorescences in the surroundings of treated colonies. It is proposed that the joint administration of synthetic mimic odors of the target crop in combination with these nonsugar compounds, which act as memory enhancers, could further improve bee pollination efficiency in commercial crops.

## Results

### Colony foraging activity

**Figure 1:**
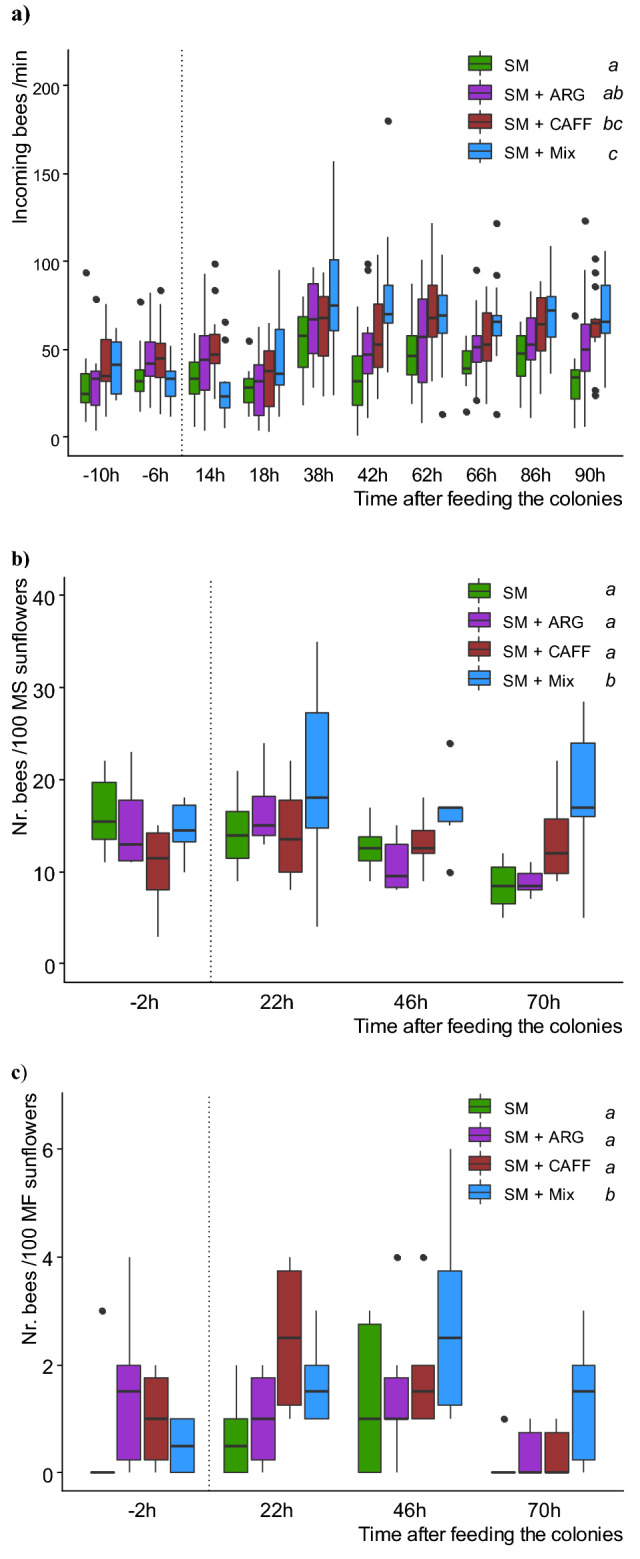
Effect of a Sunflower Mimic (SM) combined with nectar’s nonsugar compounds on honeybees foraging. Colonies providing pollination services in an oilseed sunflower hybrid plot were fed: SM-scented food (as control), and SM-scented food supplemented with either caffeine (SM + CAFF), l-arginine (SM + ARG), or a mixture of both compounds (SM + Mix). (**a**) Colony activity: Rate of incoming bees before (− 10, − 6 h) and after the offering of the treatments (up to 90 h) (N = 15 colonies per treatment). (**b**) Honeybees’ density on male sterile (MS) sunflower inflorescences in the surroundings of treated colonies (N = 6 linear transects of 100 open inflorescences per treatment). (**c**) Honeybees’ density on male fertile (MF) sunflower inflorescences in the surroundings of treated colonies (N = 6 linear transects of 100 open inflorescences per treatment). Boxplots show the median and IQR, with whiskers showing the maximum value within 1.5 IQR, and individual points mark values outside this range. The vertical dotted line indicates the administration of the treatments. Different letters indicate significant differences (p < 0.05) for each treatment as assessed with post hoc comparisons.

To evaluate whether the circulation of scented food supplemented with nonsugar compounds (NSC) inside the hives altered foraging activity, we stimulated bee colonies offering SM-scented food (SM) as control, or SM-scented food supplemented with caffeine (SM + CAFF), arginine (SM + ARG) or a mixture of both compounds (SM + Mix). Our data show that most colonies increased their foraging activity after the offering of scented food, with a significant treatment effect (Fig. 1a; LRT = 21.313; df = 3; p < 0.001, minimal adequate model: Nr incoming bees/min ~ treatment + time + (1|hive); see Supplementary Table S1). The increase in incoming rates was stronger in colonies fed SM + Mix than in control colonies (p < 0.001; see Supplementary Table S2) and in colonies fed SM + ARG (p = 0.041). While the incoming rates of the first group did not differ from those observed in colonies fed SM + CAFF (p > 0.05), the administration of SM + CAFF resulted in an increase in the number of incoming bees compared to control colonies (p = 0.001). However, the incoming rates did not differ between control colonies and those fed SM + ARG, nor did they vary between colonies receiving the individual NSC (p > 0.05). It is not trivial to clarify that before the administration of the treatments, colonies showed similar levels of foraging activity (p > 0.05; see Supplementary Table S1).

### Honeybees foraging on the crop

We evaluated to what extent the offering of SM-scented food affected colony foraging intensity on both sunflower parental lines (MS and MF). Density of foragers measured in MS and MF sunflowers (Fig. 1b,c, respectively) showed that more bees were found in the surroundings of SM + Mix-treated colonies than in the surroundings of other treated colonies (LRT = 24.003; df = 3; p < 0.001 minimal adequate model: Nr bees per transect ~ treatment + parental line + time + (1|transect) + offset (log(blooming)); see Supplementary Table S1). Statistically similar numbers of bees were counted in the surroundings of control colonies and the ones fed the individual SC (Control vs SM + CAFF, Control vs SM + ARG, SM + CAFF vs SM + ARG, p > 0.05; see Supplementary Table S2). In this case, there were also no differences between treatments, prior to the offering of the scented food (p > 0.05; see Supplementary Table S1).

### Crop yield

**Figure 2:**
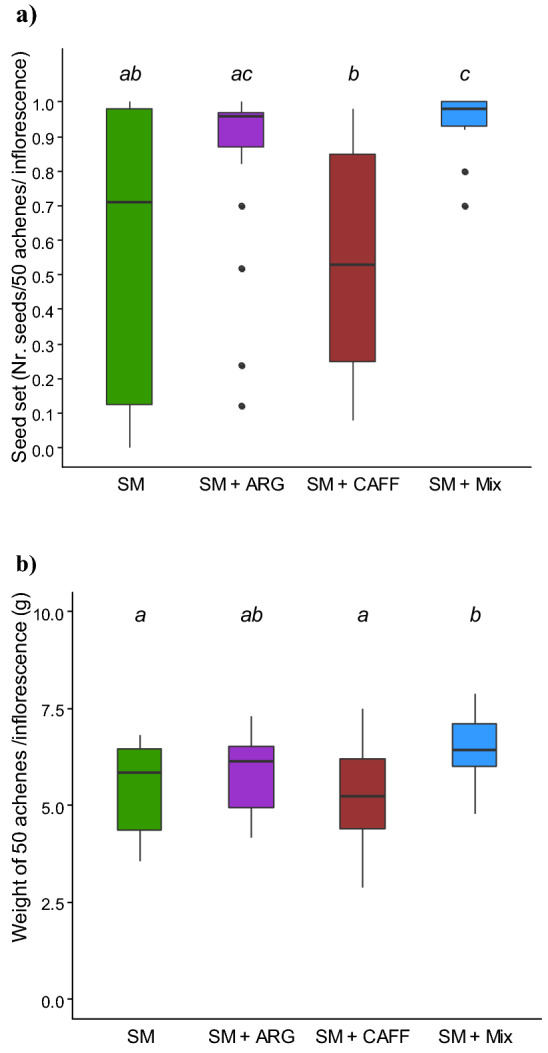
Effect of a Sunflower Mimic (SM) combined with nectar’s nonsugar compounds on crop yield. Colonies providing pollination services in an oilseed sunflower hybrid plot were fed: SM-scented food (as control), and SM-scented food supplemented with either caffeine (SM + CAFF), l-arginine (SM + ARG), or a mixture of both compounds (SM + Mix). (**a**) Sunflower seed set was calculated as the number of filled achenes (seeds) in 50 achenes randomly harvested per inflorescence (N = 20 inflorescences per treatment). (**b**) Weight of 50 achenes randomly harvested per inflorescence (N = 20 inflorescences per treatment). Boxplots show the median and IQR, with whiskers showing the maximum value within 1.5 IQR, and individual points mark values outside this range. Different letters indicate significant differences (p < 0.05) for each treatment as assessed with post hoc comparisons.

After the offering of SM-scented food, we evaluated changes in sunflower seed yields in the surroundings of the treated colonies (Fig. 2). Seed set was significantly different between treatments (Fig. 2a, LRT = 20.93; df = 3; p < 0.001, minimal adequate model: Nr seeds/50 achenes ~ treatment + (1|ID); see Supplementary Table S1), being higher in those inflorescences pollinated by colonies fed SM + Mix than by control colonies and by colonies fed SM + CAFF (p = 0.004 and p < 0.001, respectively; see Supplementary Table S2). While seed set resulted similar in the surroundings of colonies fed SM + Mix and those fed SM + ARG (p > 0.05), it was significantly higher in the surroundings of colonies fed SM + ARG than in proximity of those fed SM + CAFF (p = 0.029). On the other hand, there was no significant effect of the administration of individual NSC compared to the control (p > 0.05).

In addition, the weight of 50 achenes was also significantly affected by the treatment (Fig. 2b, LRT = 16.6395; df = 3; p < 0.001, minimal adequate model: Weight ~ treatment + initial head size; see Supplementary Table S1). The difference in weight is due to the proportion of filled achenes (‘seed mass’) and empty hulls, being a good indicator of yield. Seed mass was higher by 20% on inflorescences close to colonies fed SM + Mix, than on sunflowers close to control colonies (p = 0.008; see Supplementary Table S2). Also, their seed mass significantly differed from sunflowers in the proximity of colonies fed SM + CAFF (p < 0.001). However, there were no significant differences between the combined administration of both NSC compared to the solely administration of arginine (p > 0.05). On the other hand, seed mass resulted similar among sunflowers near control colonies and those receiving the individual NSC, arginine and caffeine, respectively (p > 0.05).

## Discussion

Our results show the long-lasting positive effect of the oral administration of scented food supplemented with a mixture of two nectar nonsugar compounds (NSC) on the foraging activity of honeybees providing pollination services to sunflower seed production systems. Feeding colonies with a sunflower mimic odor combined with the mixture of caffeine and arginine (SM + Mix) resulted in significant increases of the foraging activity compared to control colonies, both at the colony level and on the crop. Additionally, the crop yield was significantly higher on sunflowers close to colonies fed SM + Mix compared to sunflowers in the surroundings of control colonies. However, there was no discernible effect of the individual NSC neither on the honeybee foraging activity nor in the crop yield, suggesting an additive effect of both compounds.

Most colonies increased their foraging activity at the colony level after the offering of scented food, especially after 24 h. Although arginine added individually to the scented food showed a positive trend in the incoming rates of foragers after 24 h, this treatment did not differ significantly from the control. However, the administration of caffeine and the combination of caffeine and arginine resulted in a significant increase of the foraging responses compared to control colonies, which was sustained up to 90 h after treatment. Apart from higher activity at the colony, higher densities of honeybee foragers were found on sunflowers in the surroundings of colonies fed SM + Mix, regardless of the parental line, denoting the activation of foraging towards the target crop. Such results agree with the positive effect of these two compounds jointly administered on memory retention of conditioned honeybees under laboratory conditions^33^. In the mentioned study, the mixture of both NSC (in the same concentrations as considered herein) improved short and long-term olfactory memories, tested 15 min and 24 h after conditioning restrained bees. It is well-known that olfactory memories established inside the nest can bias foraging preferences towards the learned stimulus^7–12^ and recently, it has been shown that in-hive learning of a simple synthetic odorant mixture mimicking sunflower scent guided bees towards the target crop^13^. Moreover, previous laboratory-based studies have reported that caffeinated nectar biased the initial preference of generalist bees toward the target odor, even when the NSC was not present in the reward^25^. However, there are few studies evaluating the effect of NSC on the foraging behavior of bees at individual and colony level in the field and over a longer time scale. Couvillon and colleagues^24^ reported augmented individual foraging frequency and recruitment behavior towards an artificial feeder offering a caffeinated sucrose solution as reward. In the present field study, the offering of in-hive treatments enhanced foraging activity, measured both at the colony level and on the crop, suggesting that the addition of field realistic doses of these combined NSC with a floral odor can promote stable odor-specific memories for up to 3 days.

A higher foraging activity could indirectly promote the transfer of pollen from male fertile (MF) to male sterile (MS) sunflowers and translate into seed production. In the studied system, higher yields were obtained in the surroundings of SM + Mix-treated colonies. Sunflowers pollinated by these colonies set almost twice as many seeds as the ones registered in the surroundings of control colonies. Such higher proportion of filled achenes translated into a 20% increase in the weight of achenes (i.e., seed mass) compared to control plants. These results are consistent with the higher yields previously reported for different sunflower hybrids in plots pollinated by colonies fed with SM-scented food^13^. The results herein presented show that such increase in crop yield could be further augmented by the addition of combined NSC.

Overall, our results demonstrate a significant effect of the mixture of both NSC on the foraging activity of honeybees and on the yield, while the effect of each one on its own was less evident and depended on the variable measured. Although the sole administration of caffeine boosted foraging activity at the colony level, it had no significant effect on the number of bees visiting the crop nor did it result in higher yields. Such results suggest an additive effect of both compounds and might relate to the different metabolic pathways involved. On the one hand, while caffeine has been shown to enhance the activation of mushroom body neurons involved in high-order memory formation processes^23^, the oxidation of arginine produces NO, which in turn participates in LTM processes (≥ 1 day)^30,31,34^. On the other hand, while the consumption of caffeinated nectar has been shown to activate foraging responses within a few hours^24^, one would expect a deferred effect of arginine consistent with NO-dependent LTM processes and with previous results showing that the administration of arginine in the sucrose reward was not enough to enhance memory retention by 24 h, though the combined action of both NSC did^33^. Although further studies are needed to thoroughly understand the underlying mechanisms of the interaction of these two compounds, our results show a clear effect of their combination across all variables measured. Also, caffeine and NO have been reported to participate in honeybee immune responses^35,36^ which might assist colonies to sustain a numerous foraging workforce.

Given the fact that honeybees remain the main managed pollinator of crops around the world^1^, any strategy that can improve their efficiency in agricultural settings can help to facilitate crop pollination and as a result may increase global food production. Although our data are specific to sunflower hybrid seed production, it is likely that the offering of a mimic odor combined with nectar NSC can enhance honeybee pollination efficiency for other pollinator-dependent crops having separate male and female flowers or species with strong self-incompatibility systems^37^. Also, the integration of knowledge about honeybees’ cognitive processes as part of pollinator management could attenuate the dependence on honeybees by reducing the densities of beehives required for pollination services. Finally, to our knowledge, this is the first report of the combined effects of NSC naturally found in nectars, which act at the behavioral social level, facilitating the learning of odorant mixtures within the colony and promoting stable long-term social responses with potential consequences in crop production. It is worth mentioning that because the mimic odor and the NSC considered are natural compounds frequently found in flowers, offered at a very low concentration, their handling is safe and, as well as any waste generated during the application. On the whole, these findings support the use of mimic odors together with combined nectar secondary compounds as part of a precision pollination strategy in pollinator-dependent agroecosystems.

## Methods

### Study site, animals, and chemical compounds

This study was performed during the sunflower (*Helianthus annuus*) blooming season in 2021, in a plantation for hybrid seed production (oilseed cultivar) located near Coronel Pringles (38° 07′ 03.3″ S, 61° 26′ 50.2″ W), in the Austral biogeographic district of the Pampas^38^, an intensively managed region in Argentina. The sunflower plantation was immersed in an agricultural landscape, mostly dominated by productive fields, grown mainly with cereals such as soybean, maize and wheat, with few semi-natural areas (see Supplementary Fig. S1). The selected field was a circular 52-ha plot with a center-pivot irrigation system, which ensures uniformity in the amount of water application. The arrangement of the male fertile (MF) and male sterile (MS) lines of the sunflower cultivar consisted of four MF lines every fourteen MS ones, alternated in this proportion throughout the field width. The adjacent plots were grown with soybean (which was not in bloom at the time of the study) or were covered with crop’s stubble.

Commercial *Apis mellifera* Langstroth hives (about 20,000 workers) were introduced in the field to provide pollination services at a stocking density of 3 hives per hectare, which is the suggested stocking rate for sunflower seed production^39^. A total of 80 experimental honeybee hives, distributed around the field in groups of 20 hives each, were selected and stimulated (see treatments below; see Supplementary Fig. S2). Beekeepers were informed about the study and provided consent for honeybee manipulation. Previous studies in sunflower plantations in this intensively managed region have documented the dominance of honey bees as pollinators, accounting for more than 90% of sunflower visits^40,41^.

The chemical compounds used to prepare the different treatments administered to the beehives were acquired in Sigma-Aldrich, Steinheim, Germany. The synthetic mixture (Sunflower mimic) is composed by sabinene (48%), (−)-beta-pinene (40%) and limonene (12%)^42^. The concentrations of caffeine and arginine considered are within or below the natural range found in nectars^33^. These authors found that such concentrations (see section below) positively affected olfactory memories in honeybees without compromising their survival.

### Colony stimulation

Scented food was obtained by diluting 50 μL of synthetic mixture (sunflower mimic, SM) per liter of sucrose solution (50% weight/weight, henceforth: w/w). Four treatments were considered: SM-scented food (as a control, N = 20 hives), SM-scented food supplemented with caffeine [0.15 mM] (SM + CAFF, N = 20 hives), SM-scented food supplemented with arginine [0.03 mM] (SM + ARG, N = 20 hive), or SM-scented food supplemented with a mixture of both (SM + Mix, N = 20 hives). Colonies were fed in situ by pouring 500 mL of one of the solutions, over the top of the central frames of the hives.

### Colony foraging activity

To evaluate whether the circulation of SM-scented food supplemented with SC inside the hives altered foraging activity, we compared the activity level between the colonies fed with the four treatments described above. To this end, we calculated the rate of incoming bees in 15 colonies per treatment. Incoming bees were counted for 1 min each morning (9:00 am) and each afternoon (13:00 pm) for four consecutive days. Two measurements were done before feeding the colonies (− 10, − 6 h) and eight measurements afterward (up to 90 h).

### Honeybees foraging on the crop

To test if the offering of SM-scented food positively biased honeybees’ foraging on sunflower inflorescences in the surroundings of treated colonies, we recorded the number of bees per 100 open flowering units (ofu) present along linear transects in between the field rows, considering both parental lines (6 transects in MS lines and 6 MF lines/each treatment). Measurements were done once before feeding the colonies (− 2 h) and three times afterward (up to 70 h).

### Crop yield

Finally, we evaluated the effects of beehive stimulation on crop yields. We labeled 20 sunflowers capitula (head) per treatment, at 20 m from the colonies fed with different treatments, and calculated flower head size by measuring the head diameter across the outermost whorl of florets. Afterwards, approximately 3 weeks after bloom, capitula were sampled when seeds were mature, black and ready to harvest. For each flower head, we recorded: sunflower seed set, calculated as the number of filled achenes (seeds) in 50 achenes randomly harvested per inflorescence; and weight of 50 achenes randomly harvested per inflorescence.

The distance between the groups of hives (receiving the different treatments) ranged from 260 to 430 m. Although bees from different colonies could not be identified, the probability that the marked sunflowers were visited by bees from the other treated hives (more distant) is lower, given the abundance of sunflowers and the copious amount of nectar offered, the lack of alternative flora nearby, and because the numbers of bees on the crop and the seed production decreased as distance from the hives increased in other studied plantations^43^. Johannsmeier and Mostert^44^ observed that honeybee foragers started decreasing in number after 250 m, suggesting that they prefer near distances when the same profitable resource is available.

### Statistical analysis

All statistical tests were performed with R v3.6.2^45^, using the glmmTMB package^46^. We considered the use of generalized linear and mixed-effect models (GLM and GLMM) because these models allow analyzing response variables whose errors are not normally distributed, avoiding the transformation of the response variable or the adoption of non-parametric methods^47^. All models were inspected for over-/under dispersion, zero inflation and distribution of the residuals. Scaled residuals were simulated from the fitted model using the DHARMa package^48^. Significance of the different terms in models was tested starting from the higher-order terms model using likelihood ratio tests (LRT) with anova function to compare between nested models^49^. Non-significant terms (p > 0.05) were removed (see Supplementary Table S1). Post hoc comparisons across treatments were performed with the emmeans package^50^ and Tukey method was used to adjust for multiple testing (see Supplementary Table S2).

To test for differences among incoming rates, we proposed a GLMM with a negative binomial error distribution or a Conway–Maxwell Poisson error distribution, before and after hives feeding, with time (a two or eight-level factor) and treatment (a four-level factor) included as fixed effects and hive as random effect. We considered a negative binomial error distribution to account for the overdispersion of the data.

To test for differences between bee densities on the crop, we proposed a GLMM with a negative binomial error distribution, with parental line (a two-level factor) and treatment (a four-level factor) included as fixed effects. In both cases, before and after hives feeding, we included the log-transformed percentage of blooming as an offset, to correct the number of events for an estimate of population size. In addition, in the post-stimulation model, we included the transect as random factor to account for the repeated measurements and time (a three-level factor) as a fixed effect. We considered a negative binomial error distribution to account for the overdispersion of the data.

To evaluate the effect of beehive stimulation on seed set, we proposed a GLMM with a binomial error distribution, with treatment (a four-level factor) as fixed effect, and capitulum identity as an observation level random effect (OLRE), to account for the overdispersion of the data. To test for differences among the weight of 50 achenes, we proposed a GLM following a Gaussian error distribution, with treatment (a four-level factor) as fixed effect and initial head size as covariate. We initially included a one-way interaction (treatment × initial head size) but since it was non-significant (p > 0.05), we proposed an additive model (see Supplementary Table S1).

### Statement on the collection of plant or seed specimens

The producers granted permission for plant and seed measurements, which were done in situ. All methods and assays performed in this field study comply with national legislation and guidelines of the University of Buenos Aires and CONICET-Argentina.

## Supplementary Information

Supplementary Information.

## Data Availability

The datasets generated for this study are available on request to the corresponding author.
