# Supplementary material for: Learning of a mimic odor combined with nectar nonsugar compounds enhances honeybee pollination of a commercial crop
Source: Sci Rep. 2021 Dec 14;11:23918. doi: 10.1038/s41598-021-03305-9 (PMC8671565; doi:10.1038/s41598-021-03305-9)
Supplement: Supplementary file 1 — Supplementary Information. [file 41598_2021_3305_MOESM1_ESM.doc]

Supplementary Material

**Learning of a mimic odor combined with nectar nonsugar compounds enhances honeybee pollination of a commercial crop**

M. Cecilia Estravis-Barcala^1,2#^, Florencia Palottini^1,2#^, and Walter M. Farina^1,2^*

*^1^Laboratorio de Insectos Sociales, Departamento de Biodiversidad y Biología Experimental, Facultad de Ciencias Exactas y Naturales, Buenos Aires, Argentina.*

*^2^Instituto de Fisiología, Biología Molecular y Neurociencias (IFIBYNE), CONICET-Universidad de Buenos Aires, Buenos Aires, Argentina.*

# These authors contributed equally to this work

*Corresponding author: Email: [walter@fbmc.fcen.uba.ar](mailto:walter@fbmc.fcen.uba.ar)

**Supplementary Figure S1**. Location of the sunflower plantation in the Austral biogeographic district of the Pampas, an intensively managed region in Argentina (38°07'03.3"S, 61°26'50.2"W). S, sunflower plot; M, premises and machinery area; R, route. (Map data: Google, CNES / Airbus, Maxar Technologies)

**Supplementary Figure S2**. Distribution of the experimental honeybee colonies (N = 80) in the sunflower plot. Hexagons represent groups of 20 hives and legends indicate the four treatments detailed in Methods. (Map data: Google, CNES / Airbus)

**Supplementary Table S1**. Set of variables considered in the generalized linear (mixed) models proposed for colony activity, honey bee foraging on the crop and crop yield. Values in bold represent p-values<0.05.

| Section and Model | Coefficient | Std. Error | Z value | p-value |
| --- | --- | --- | --- | --- |
| **Colony activity** | | | | |
| *a) Before treatment*  Model (Negative Binomial): Nr incoming bees /min ~ treatment + time + (1|hive) | | | | |
| Fixed effect:  Intercept  Treatment (SM + ARG)  Treatment (SM + CAFF)  Treatment (SM + Mix)  Time (-6h) | 3.39  0.11  0.31  0.11  0.13 | 0.11  0.15  0.14  0.14  0.07 | 30.66  0.77  2.12  0.78  1.68 | **<2e-16**  0.442  **0.034**  0.433  0.094 |
| *b) After treatment*  Model (Conway-Maxwell-Poisson): Nr incoming bees /min ~ treatment + time + (1|hive) | | | | |
| Fixed effect:  Intercept  Treatment (SM + ARG)  Treatment (SM + CAFF)  Treatment (SM + Mix)  Time (+18h)  Time (+38h)  Time (+42h)  Time (+62h)  Time (+66h)  Time (+86h)  Time (+90h) | 3.34  0.22  0.39  0.49  -0.13  0.52  0.36  0.45  0.32  0.41  0.34 | 0.09  0.10  0.10  0.10  0.08  0.07  0.07  0.07  0.07  0.07  0.07 | 36.83  2.09  3.75  4.74  -1.66  7.65  5.15  6.45  4.54  5.90  4.75 | **<2e-16**  **0.037**  **1.8e-04**  **2.1e-06**  0.097  **2.0e-14**  **2.6e-07**  **1.2e-10**  **5.5e-06**  **3.6e-09**  **2.1e-06** |
| **Honey bee foraging on the crop** | | | | |
| *a) Before treatment*  Model (Negative Binomial):  Nr bees per transect ~ treatment + parental line + offset (log(blooming)) | | | | |
| Fixed effect:  Intercept  Treatment (SM + ARG)  Treatment (SM + CAFF)  Treatment (SM + Mix)  Parental line (MF) | -0.48  0.30  -0.41  0.11  -1.58 | 0.19  0.27  0.28  0.27  0.26 | -2.51  1.09  -1.48  0.41  -6.04 | **0.012**  0.276  0.138  0.681  **1.5e-09** |
| *b) After treatment*  Model (Negative Binomial):  Nr bees per transect ~ treatment + parental line + time + (1|transect) + offset (log(blooming)) | | | | |
| Fixed effect:  Intercept  Treatment (SM + ARG)  Treatment (SM + CAFF)  Treatment (SM + Mix)  Parental line (MF)  Time (+46h)  Time (+70h) | -1.29  0.20  0.27  0.89  -0.77  -0.28  -0.80 | 0.15  0.18  0.18  0.17  0.14  0.12  0.12 | -8.75  1.15  1.56  5.14  -5.38  -2.37  -6.42 | **<2e-16**  0.251  0.121  **2.7e-07**  **7.4e-08**  **0.018**  **1.3e-10** |
| **Crop yield** | | | | |
| *a) Seed set*  Model (Binomial): Nr seeds /50 achenes ~ treatment + (1|ID) | | | | |
| Fixed effects:  Intercept  Treatment (SM + ARG)  Treatment (SM + CAFF)  Treatment (SM + Mix) | 0.98  1.52  -0.65  3.21 | 0.57  0.79  0.77  0.90 | 1.73  1.92  -0.84  3.57 | 0.084  0.055  0.401  **3.6e-04** |
| *b) Weight of 50 achenes*  Model^#1^ (Gaussian): Weight ~ treatment + initial head size | | | | |
| Fixed effects:  Intercept  Treatment (SM + ARG)  Treatment (SM + CAFF)  Treatment (SM + Mix)  Initial head size | 4.65  0.37  -0.20  1.07  0.008 | 0.51  0.33  0.33  0.32  0.005 | 9.04  1.12  -0.62  3.32  1.65 | **<2e-16**  0.261  0.536  **9.1e-04**  0.099 |
| *#1, the model initially included a one-way interaction but it was non-significant (LR = 1.70, p = 0.6376).* | | | | |

**Supplementary Table S2**. *Post hoc* comparisons among treatments in colony activity, honeybee densities on the crop yield. t ratio values (below diagonal) and p-values (above diagonal) obtained from pairwise comparisons between the four treatments: SM-scented food as a control (SM), SM-scented food supplemented with caffeine [0.15mM] (SM + CAFF), SM-scented food supplemented with arginine [0.03mM] (SM + ARG), or SM-scented food supplemented with a mixture of both (SM + Mix). P-values were adjusted with Tukey method for multiple testing. Values in bold represent p-values<0.05.

| **Colony activity** | | | | |
| --- | --- | --- | --- | --- |
| **Treatment** | SM | SM + ARG | SM + CAFF | SM + Mix |
| SM |  | 0.1577 | **0.0011** | **<0.0001** |
| SM + ARG | -2.09 |  | 0.3450 | **0.0409** |
| SM + CAFF | -3.75 | -1.66 |  | 0.7531 |
| SM + Mix | -4.74 | -2.65 | -0.99 |  |
| **Honeybees foraging on the crop** | | | | |
| **Treatment** | SM | SM + ARG | SM + CAFF | SM + Mix |
| SM |  | 0.6612 | 0.4100 | **<0.0001** |
| SM + ARG | -1.15 |  | 0.9797 | **0.0006** |
| SM + CAFF | -1.55 | -0.39 |  | **0.0018** |
| SM + Mix | -5.14 | -4.00 | -3.69 |  |
| **Crop yield:**  **a) Seed set** | | | | |
| **Treatment** | SM | SM + ARG | SM + CAFF | SM + Mix |
| SM |  | 0.2297 | 0.8354 | **0.0037** |
| SM + ARG | -1.92 |  | **0.0289** | 0.2425 |
| SM + CAFF | 0.84 | 2.85 |  | **0.0002** |
| SM + Mix | -3.57 | -1.89 | -4.40 |  |
| **b) Weight of 50 achenes** | | | | |
| **Treatment** | SM | SM + ARG | SM + CAFF | SM + Mix |
| SM |  | 0.6762 | 0.9259 | **0.0077** |
| SM + ARG | -1.12 |  | 0.2899 | 0.1326 |
| SM + CAFF | 0.62 | 1.78 |  | **0.0009** |
| SM + Mix | -3.32 | -2.20 | -3.99 |  |
